# New records of stoneflies (Insecta, Plecoptera) from the Greater Khingan Mountains, China

**DOI:** 10.3897/BDJ.13.e151489

**Published:** 2025-04-16

**Authors:** Ya-Fei Zhu, Abdur Rehman, Xiao Yang, Qing-Bo Huo, Liang-Liang Zeng, Valentina A. Teslenko, Yu-Zhou Du

**Affiliations:** 1 College of Plant Protection & Institute of Applied Entomology，Yangzhou University, Yangzhou, China College of Plant Protection & Institute of Applied Entomology，Yangzhou University Yangzhou China; 2 Federal Scientific Center of the East Asia Terrestrial Biodiversity, Far Eastern Branch, Russian Academy of Sciences (FSC EATB FEB RAS), 690022, Vladivostok, Russia Federal Scientific Center of the East Asia Terrestrial Biodiversity, Far Eastern Branch, Russian Academy of Sciences (FSC EATB FEB RAS), 690022 Vladivostok Russia; 3 Joint International Research Laboratory of Agriculture and Agri-Product Safety, the Ministry of Education, Yangzhou University, Yangzhou, China Joint International Research Laboratory of Agriculture and Agri-Product Safety, the Ministry of Education, Yangzhou University Yangzhou China

**Keywords:** Plecoptera, new recorded genus, new recorded species, China

## Abstract

**Background:**

Plecoptera (stoneflies) are an ancient order of aquatic insects that serve as vital bioindicators in freshwater ecosystems. This study focuses on the Greater Khingan Mountains in north-eastern China, a biodiversity hotspot with limited stonefly research. By documenting new genera and species records, it expands our knowledge of Chinese Plecoptera. The findings enhance biogeographical and ecological insights into this underexplored region.

**New information:**

This paper records five families, six genera and seven species of stoneflies from the Greater Khingan Mountains, among them *Diura* Billberg, 1820 and *Megarcys* Klapálek, 1912 as new Chinese-recorded genera and seven species recorded for the first time in China, including *Isocapniakudia* Ricker, 1959, *Paraleuctrazapekinae* Zhiltzova, 1974, *Nemouraarctica* Esben-Petersen, 1910, *Nemourasirotskii* Teslenko, 2018, *Diuramajuscula* (Klapálek, 1912), *Megarcysochracea* (Klapálek, 1912) and *Utaperlalepnevae* (Zhiltzova, 1970). The research materials were from the northern part of the Greater Khingan Mountains in north-eastern China, including parts of Heilongjiang Province and Inner Mongolia Autonomous Region.

## Introduction

The Greater Khingan Mountains is located in the north-eastern part of China, starting from the banks of the Heilongjiang River in the north and ending in the upper valley of the Xilamulun River in the south (40°59′N~53°33′N, 115°5′E~125°16′E, with a length of about 1400 km and a width ranging from 200 to 300 km). The Greater Khingan Mountains area is adjacent to the Russian Far East and Mongolia. Administratively, it encompasses the eastern part of the Inner Mongolia Autonomous Region (including Hulunbuir City, Hinggan League, Tongliao City and Chifeng City), as well as the north-western part of Heilongjiang Province (Da Hinggan Ling Prefecture). There are multiple hills and branches of streams on the Greater Khingan Mountains, making it an ideal habitat for stoneflies.

Since the last century, there have been relatively few records of stoneflies in the north-eastern region of China (Nelson and Hanson 1969, Yang and Yang 1996, Li et al. 2015a, Li et al. 2015b, Chen and Du 2016, Yang and Li 2018, Huo et al. 2020, Huo et al. 2022, Shi et al. 2022) and there has been a lack of systematic investigation and research. Nelson and Hanson (1969) described *Utaperlaorientalis* Nelson & Hanson, 1969, the first stonefly species with a type locality in the Greater Khingan Mountains. There were no reports of Plecoptera in the Greater Khingan Mountains for a long period thereafter. Recently, we conducted a continuous survey of rivers (including GanHe River, Pangu River, Huma River, Duobukuer River and Heilongjiang River) in the northern part of the Greater Khingan Mountains. In this paper, two genera and seven species from this region are recorded for the first time in China.

## Materials and methods

Specimens were collected by hand, sweep net and light trap and preserved in 75% ethanol. Abdominal segments of specimens were examined and illustrated by KEYENCE VHX-5000 and optimised by Adobe Photoshop CS6. The materials are deposited in the Insect Collection of Yangzhou University (ICYZU), Jiangsu Province, China.

## Taxon treatments

### Isocapnia kudia

Ricker, 1959

6B7CDC49-4081-5ABD-9974-957A8C3758C2

Isocapnia kudia Ricker, 1959: 650

#### Materials

- **Type status:** Other material. **Occurrence:** recordedBy: Ya-Fei Zhu & Xiao Yang; individualCount: 1; sex: male; lifeStage: adult; occurrenceStatus: present; disposition: ICYZU; occurrenceID: 835990E5-B8A6-5C49-AFFF-5F796C540B97; **Taxon:** scientificName: *Isocapniakudia* Ric*k*er, 1959; kingdom: Animalia; phylum: Arthropoda; class: Insecta; order: Plecoptera; family: Capniidae; genus: Isocapnia ; specificEpithet: *kudia*; taxonRank: species; taxonomicStatus: Valid; **Location:** continent: Asia; country: China; countryCode: CN; stateProvince: Heilongjiang; locality: Da Hinggan Ling Prefecture, Jiagedaqi District; maximumElevationInMeters: 451; verbatimLatitude: 50.403951; verbatimLongitude: 124.120544; **Identification:** identifiedBy: Xiao Yang; **Event:** year: 2023; month: 6; day: 3; **Record Level:** language: en; basisOfRecord: PreservedSpecimen

#### Diagnosis

**Figure 1.**
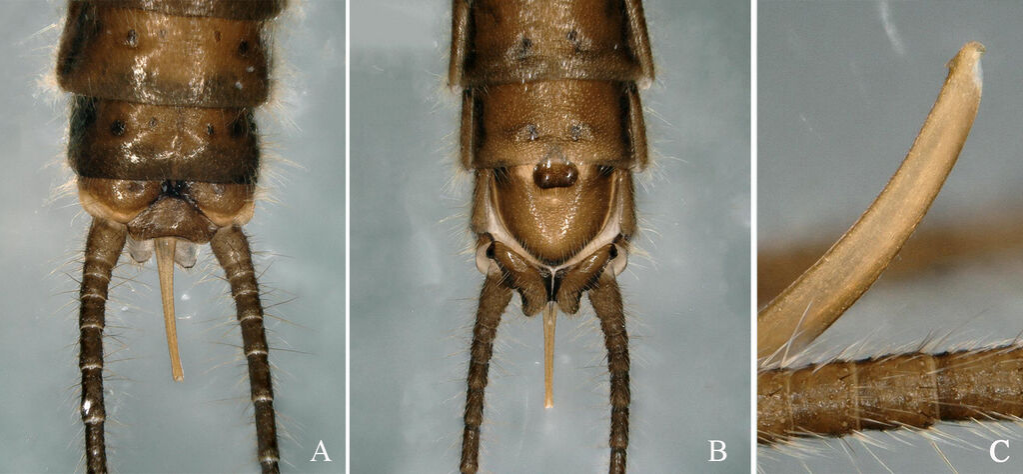
*Isocapniakudia* Ricker, 1959. **A-B** male terminalia, dorsal, ventral; **C** epiproct, lateral view.

Epiproct in lateral view almost straight, directed upwards, slightly thickened in the middle, its tip with a small tooth in front of the apex (Fig. 1A-C).

#### Distribution

Russia, Transbaikalia, Far East (Teslenko and Zhiltzova 2009). The species is recorded for China for the first time.

### Paraleuctra zapekinae

Zhiltzova, 1974

F3E3C384-3D82-585B-AF5C-E678462E7ADC

Paraleuctra zapekinae Zhiltzova, 1974: 360

#### Materials

- **Type status:** Other material. **Occurrence:** individualCount: 10; sex: male; lifeStage: adult; occurrenceStatus: present; disposition: ICYZU; occurrenceID: 9CE13161-BA4E-5C46-8FA3-D235BCA28FE1; **Taxon:** scientificName: *Paraleuctrazapekinae* Zhiltzova, 1974; kingdom: Animalia; phylum: Arthropoda;; class: Insecta; order: Plecoptera; family: Leuctridae; genus: Paraleuctra; specificEpithet: *zapekinae*; taxonomicStatus: Valid; **Location:** continent: Asia; country: China; countryCode: CN; stateProvince: Heilongjiang; locality: Da Hinggan Ling Prefecture, Xinlin District, Dawusu town; maximumElevationInMeters: 471; verbatimLatitude: 51.768247; verbatimLongitude: 124.512166; **Identification:** identifiedBy: Xiao Yang; **Event:** year: 2023; month: 6; day: 6; **Record Level:** language: en; institutionCode: ICYZU

#### Diagnosis

**Figure 2.**
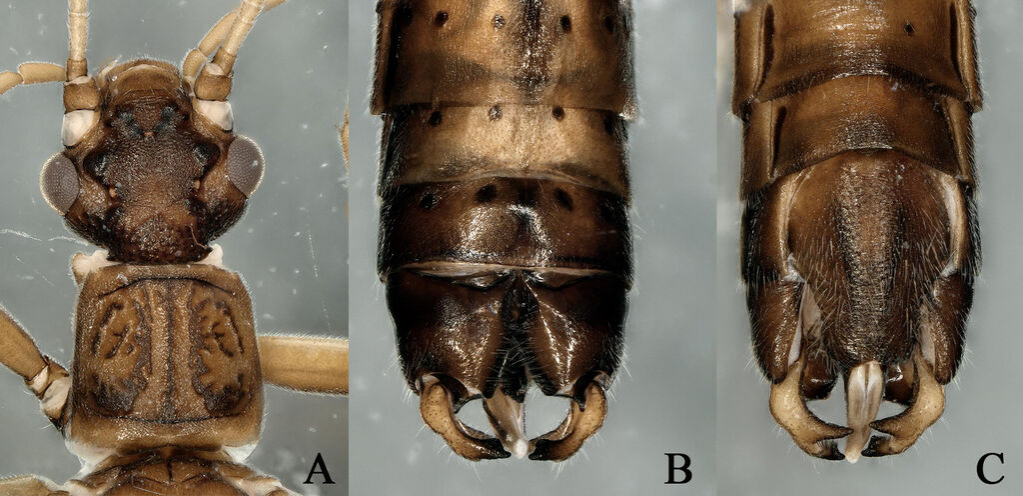
*Paraleuctrazapekinae* Zhiltzova, 1974. **A** male head and pronotum; **B-C** male terminalia, dorsal and ventral view.

Male cerci are slightly swollen at the base, sharply curved and directed medially with their apices; the ventral tooth of the cerci without an additional tooth and the dorsal tooth posteriorly has a short tooth and bluntly bifurcated apex (Fig. 2A-C).

#### Distribution

Russia, Siberia, Far East (Teslenko and Zhiltzova 2009). Mongolia (DeWalt et al. 2024). The species is recorded for China for the first time.

### Nemoura arctica

Esben-Petersen, 1910

9CD0D1B0-75B4-5480-A6CF-A8396D5A4EC6

Nemoura arctica Esben-Petersen, 1910: 85.
Nemoura trispinosa Claassen, 1923:289.

#### Materials

- **Type status:** Other material. **Occurrence:** recordedBy: Ya-Fei Zhu & Xiao Yang; individualCount: 1; sex: male; lifeStage: adult; occurrenceStatus: present; occurrenceID: 2EE0772D-FF21-5E04-81B9-84BFC182AF62; **Taxon:** scientificName: *Nemouraarctica* Esben-Petersen, 1910; kingdom: Animalia; phylum: Arthropoda; class: Insecta; order: Plecoptera; family: Nemouridae; genus: Nemoura ; specificEpithet: *arctica*; taxonomicStatus: valid; **Location:** continent: Asia; country: China; countryCode: CN; stateProvince: Inner Mongolia Autonomous Region; locality: HulunBuir city, Genhe city, Jinhe town; maximumElevationInMeters: 791; verbatimLatitude: 51.331111; verbatimLongitude: 121.491944; **Identification:** identifiedBy: Ya-Fei Zhu & Valentina A. Teslenko; **Event:** year: 2023; month: 5; day: 27; **Record Level:** language: en; institutionCode: ICYZU

#### Diagnosis

**Figure 3.**
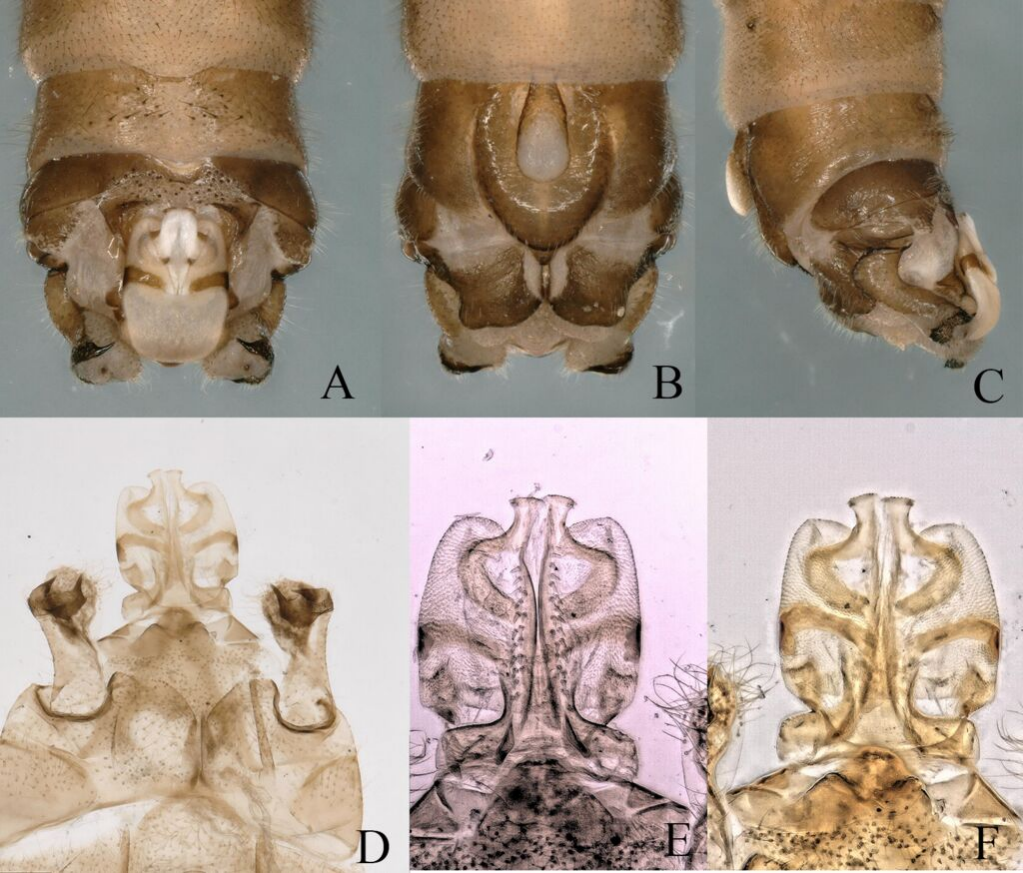
*Nemouraarctica* Esben-Petersen, 1910. **A-C** male terminalia, dorsal, ventral and lateral view; **D** epiproct dorsal and cerci, ventral view; **E-F** epiproct, ventral and dorsal view.

Males exhibit consistency with epiproct shape and characteristics across the Holarctic. The apical sclerite terminates laterally, bearing two short, thick spines. Male cerci are sclerotized laterally and terminate typically in a pair of appressed spines; they also have an outer spine (Fig. 3A-F).

#### Distribution

Canada, USA, and Europe, including Estonia, Finland, Latvia, Lithuania, Norway and Sweden, Mongolia (DeWalt et al. 2024). Russia, north of the European part, Siberia, Far East (Teslenko and Zhiltzova 2009). The species is recorded for China for the first time.

### Nemoura sirotskii

Teslenko, 2018

B43C684C-9057-517D-83EA-B31B066B4101

Nemoura sirotskii Teslenko 2018 in Teslenko & Boumans (2018: 154)

#### Materials

- **Type status:** Other material. **Occurrence:** recordedBy: Ya-Fei Zhu & Xiao Yang; individualCount: 1; sex: male; lifeStage: adult; occurrenceStatus: present; occurrenceID: DBDA7C8D-CC95-52DA-BF2B-BC5A399826C4; **Taxon:** scientificName: *Nemourasirotskii* Teslenko, 2018; kingdom: Animalia; phylum: Arthropoda; class: Insecta; order: Plecoptera; family: Nemouridae; genus: Nemoura ; specificEpithet: *sirotskii*; **Location:** continent: Asia; country: China; countryCode: CN; stateProvince: Heilongjiang; locality: Da Hinggan Ling Prefecture, Mohe county, Beiji town, Beihong Village; maximumElevationInMeters: 287; verbatimLatitude: 53.330427; verbatimLongitude: 123.115030; **Identification:** identifiedBy: Ya-Fei Zhu & Valentina A. Teslenko; **Event:** year: 2023; month: 5; day: 29; **Record Level:** institutionCode: ICYZU
- **Type status:** Other material. **Occurrence:** recordedBy: Ya-Fei Zhu & Xiao Yang; individualCount: 2; sex: 2 males, 2 females; lifeStage: adult; occurrenceStatus: present; occurrenceID: 2C8C68D2-891B-57D5-9FA6-B2BCFE4C1072; **Taxon:** scientificName: *Nemourasirotskii* Teslenko, 2018; kingdom: Animalia; phylum: Arthropoda; class: Insecta; order: Plecoptera; family: Nemouridae; genus: Nemoura ; specificEpithet: *sirotskii*; **Location:** continent: Asia; country: China; countryCode: CN; stateProvince: Heilongjiang; locality: Da Hinggan Ling Prefecture, Xinlin District, Dawusu town; maximumElevationInMeters: 471; verbatimLatitude: 51.768247; verbatimLongitude: 124.512166; **Identification:** identifiedBy: Ya-Fei Zhu & Valentina A. Teslenko; **Event:** year: 2023; month: 6; day: 6; **Record Level:** institutionCode: ICYZU

#### Diagnosis

**Figure 4.**
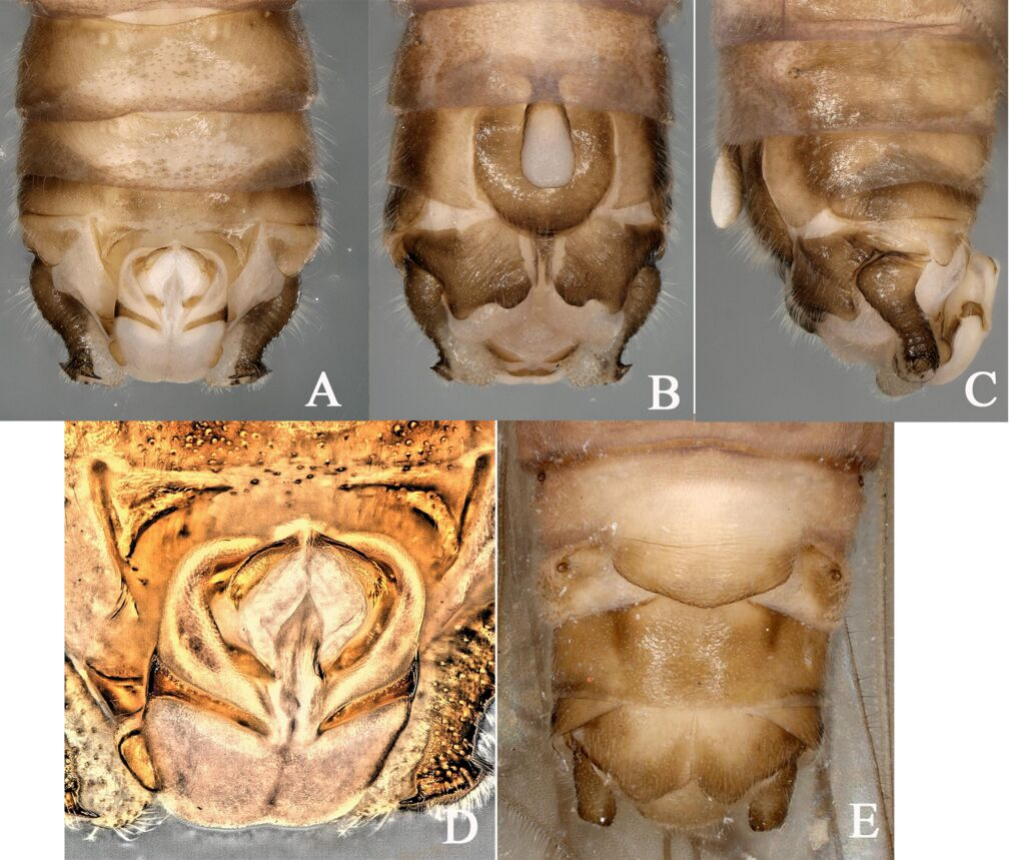
*Nemourasirotskii* Teslenko, 2018. **A-C** male terminalia, dorsal, ventral and lateral view; **D** epiproct, dorsal view; **E** female terminalia, ventral view.

Male epiproct is oval dorsally, slightly widening in the anterior half, becoming narrow basolaterally. Paired lateral arms of the dorsal sclerite directed obliquely down towards the middle, nearly touching and sclerotized; the paired dorsal folds deep and covered with comb-like scales. Each apical sclerite of the epiproct is elongated and flat-lying, with the base extending beyond the dorsal folds, directed obliquely downward, slightly widened at the apex and the edge of the distal apex is rounded and rough, bearing fine comb-like scales and 3–4 stout lateral spines. Female sternum 7 extended medially, forming a well-developed, broadly rounded and swollen pregenital plate, which is slightly(?) sclerotized, overlapping sternum 8 completely and the anterior margin of sternum 9 partly (Fig. 4A-E).

#### Distribution

Russia, Far East (Teslenko and Boumans 2018). The species is recorded for China for the first time.

### Diura majuscula

(Klapálek, 1912)

AD8DC5C2-74DE-51CC-9F46-32C2876A60E5

Dictyopterygella majuscula Klapálek, 1912: 45.
Diura majuscula Zhiltzova, 1975.

#### Materials

- **Type status:** Other material. **Occurrence:** recordedBy: Ya-Fei Zhu & Xiao Yang; individualCount: 7; sex: 3 males, 4 females; lifeStage: adult; occurrenceStatus: present; occurrenceID: 350DF541-54F4-5CE1-A85F-7C19EE812579; **Taxon:** scientificName: *Diuramajuscula* (Klapálek, 1912); kingdom: Animalia; phylum: Arthropoda;; class: Insecta; order: Plecoptera; family: Perlodidae; genus: Diura; specificEpithet: *majuscula*; taxonomicStatus: valid; **Location:** continent: Asia; country: China; countryCode: CN; stateProvince: Heilongjiang; locality: Da Hinggan Ling Prefecture, Mohe county; maximumElevationInMeters: 444; verbatimLatitude: 52.974534; verbatimLongitude: 122.543546; **Identification:** identifiedBy: Qing-Bo Huo; **Event:** year: 2023; month: 5; day: 28; **Record Level:** language: en; institutionCode: ICYZU
- **Type status:** Other material. **Occurrence:** recordedBy: Ya-Fei Zhu & Xiao Yang; individualCount: 2; sex: 2 females; lifeStage: adult; occurrenceStatus: present; occurrenceID: 350DF541-54F4-5CE1-A85F-7C19EE812579; **Taxon:** scientificName: *Diuramajuscula* (Klapálek, 1912); kingdom: Animalia; phylum: Arthropoda;; class: Insecta; order: Plecoptera; family: Perlodidae; genus: Diura; specificEpithet: *majuscula*; taxonomicStatus: valid; **Location:** continent: Asia; country: China; countryCode: CN; stateProvince: Heilongjiang; locality: Da Hinggan Ling Prefecture, Tahe county, Pangu town; maximumElevationInMeters: 562; verbatimLatitude: 52.682158; verbatimLongitude: 123.861120; **Identification:** identifiedBy: Qing-Bo Huo; **Event:** year: 2023; month: 6; day: 1; **Record Level:** language: en; institutionCode: ICYZU
- **Type status:** Other material. **Occurrence:** recordedBy: Ya-Fei Zhu & Xiao Yang; individualCount: 14; sex: 8 males, 6 females; lifeStage: adult; occurrenceStatus: present; occurrenceID: 350DF541-54F4-5CE1-A85F-7C19EE812579; **Taxon:** scientificName: *Diuramajuscula* (Klapálek, 1912); kingdom: Animalia; phylum: Arthropoda;; class: Insecta; order: Plecoptera; family: Perlodidae; genus: Diura; specificEpithet: *majuscula*; taxonomicStatus: valid; **Location:** continent: Asia; country: China; countryCode: CN; stateProvince: Heilongjiang; locality: Da Hinggan Ling Prefecture, Tahe county; maximumElevationInMeters: 363; verbatimLatitude: 52.304958; verbatimLongitude: 124.696754; **Identification:** identifiedBy: Qing-Bo Huo; **Event:** year: 2023; month: 6; day: 6; **Record Level:** language: en; institutionCode: ICYZU
- **Type status:** Other material. **Occurrence:** recordedBy: Ya-Fei Zhu & Xiao Yang; individualCount: 4; sex: 2 males, 2 females; lifeStage: adult; occurrenceStatus: present; occurrenceID: 350DF541-54F4-5CE1-A85F-7C19EE812579; **Taxon:** scientificName: *Diuramajuscula* (Klapálek, 1912); kingdom: Animalia; phylum: Arthropoda;; class: Insecta; order: Plecoptera; family: Perlodidae; genus: Diura; specificEpithet: *majuscula*; taxonomicStatus: valid; **Location:** continent: Asia; country: China; countryCode: CN; stateProvince: Heilongjiang; locality: Da Hinggan Ling Prefecture, Mohe county, Amuer town; maximumElevationInMeters: 509; verbatimLatitude: 52.833932; verbatimLongitude: 123.173923; **Identification:** identifiedBy: Qing-Bo Huo; **Event:** year: 2023; month: 6; day: 8; **Record Level:** language: en; institutionCode: ICYZU

#### Diagnosis

**Figure 5.**
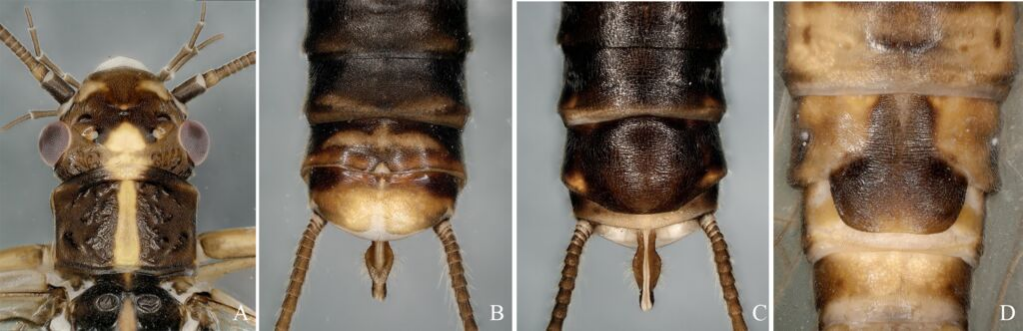
*Diuramajuscula* (Klapálek, 1912). **A** male head and pronotum; **B, C** male terminalia, dorsal and ventral view; **D** female terminalia, ventral view.

The male paraprocts are slightly swollen, laterally without a projection in front of the apex. The M-line on the head is light and clear. The female subgenital plate is strongly transverse, not tapering posteriorly, reaching half the length of the 9^th^ sternum (Fig. 5A-D).

#### Distribution

Russia, Far East (Teslenko and Zhiltzova 2009). Mongolia (DeWalt et al. 2024). The genus *Diura* Billberg, 1820 and species *D.majuscula* are recorded for China for the first time.

### Megarcys ochracea

(Klapálek, 1912)

37E62653-EC37-5808-9B6C-C3DD9CCBD8EC

Perlodes (Megarcys) ochracea Klapálek, 1912: 10.
Matsumuria sapporensis Okamoto, 1912: 16.
Perlodes yarizawana Uéno, 1931: 99.
Megarcys ochracea Kasai, 1938: 49.
Perlodes lepneva Šámal, 1939: 420.
Arcynopteryx (Megarcys) ochracea Ricker, 1952: 78.
Megarcys lepneva Illies, 1966: 370.

#### Materials

- **Type status:** Other material. **Occurrence:** recordedBy: Ya-Fei Zhu & Xiao Yang; individualCount: 6; sex: 2 males, 4 females; lifeStage: adult; occurrenceStatus: present; occurrenceID: BF80B542-C585-5297-80BB-0C53BC487D63; **Taxon:** scientificName: *Megarcysochracea* (Klapálek, 1912); kingdom: Animalia; phylum: Arthropoda; class: Insecta; order: Plecoptera; family: Perlodidae; genus: Megarcys; specificEpithet: *ochracea*; taxonomicStatus: valid; **Location:** continent: Asia; country: China; countryCode: CN; stateProvince: Heilongjiang; locality: Da Hinggan Ling Prefecture, Songlin District, Jinsong town; maximumElevationInMeters: 480; verbatimLatitude: 51.072427; verbatimLongitude: 124.194817; **Identification:** identifiedBy: Qing-Bo Huo; **Event:** year: 2023; month: 6; day: 4; **Record Level:** language: en; institutionCode: ICYZU
- **Type status:** Other material. **Occurrence:** recordedBy: Ya-Fei Zhu & Xiao Yang; individualCount: 2; sex: 1 male, 1 female; lifeStage: adult; occurrenceStatus: present; occurrenceID: BF80B542-C585-5297-80BB-0C53BC487D63; **Taxon:** scientificName: *Megarcysochracea* (Klapálek, 1912); kingdom: Animalia; phylum: Arthropoda; class: Insecta; order: Plecoptera; family: Perlodidae; genus: Megarcys; specificEpithet: *ochracea*; taxonomicStatus: valid; **Location:** continent: Asia; country: China; countryCode: CN; stateProvince: Heilongjiang; locality: Da Hinggan Ling Prefecture, Tahe county; maximumElevationInMeters: 363; verbatimLatitude: 52.304958; verbatimLongitude: 124.696754; **Identification:** identifiedBy: Qing-Bo Huo; **Event:** year: 2023; month: 6; day: 6; **Record Level:** language: en; institutionCode: ICYZU
- **Type status:** Other material. **Occurrence:** recordedBy: Ya-Fei Zhu & Xiao Yang; individualCount: 1; sex: 1 males; lifeStage: adult; occurrenceStatus: present; occurrenceID: BF80B542-C585-5297-80BB-0C53BC487D63; **Taxon:** scientificName: *Megarcysochracea* (Klapálek, 1912); kingdom: Animalia; phylum: Arthropoda; class: Insecta; order: Plecoptera; family: Perlodidae; genus: Megarcys; specificEpithet: *ochracea*; taxonomicStatus: valid; **Location:** continent: Asia; country: China; countryCode: CN; stateProvince: Heilongjiang; locality: Da Hinggan Ling Prefecture, Mohe county, Amuer town; maximumElevationInMeters: 486; verbatimLatitude: 52.832106; verbatimLongitude: 123.156700; **Identification:** identifiedBy: Qing-Bo Huo; **Event:** year: 2023; month: 6; day: 8; **Record Level:** language: en; institutionCode: ICYZU

#### Diagnosis

**Figure 6.**
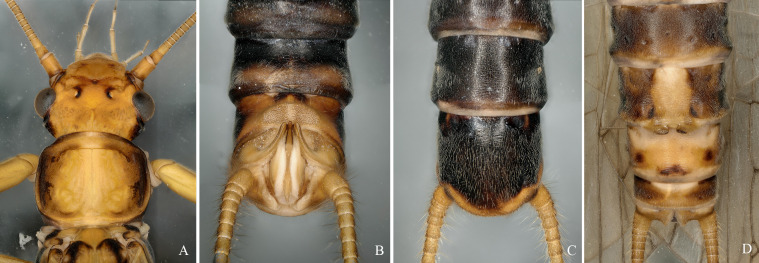
*Megarcysochracea* (Klapálek, 1912). **A** male head and pronotum; **B-C** male terminalia, dorsal and ventral view; **D** female terminalia, ventral view.

The apex of each hemitergal lobe of the male tergгum 10 is narrow laterally, bluntly rounded; the apex of the epiproct is narrow, rod-shaped; the lateral stylets are dark, tapering towards the apex and pointed. The supracoxal and submental gills are elongated. The posterior margin of the female subgenital plate is divided by a wide notch into two obliquely cut lobes directed medially backwards (Fig. 6A-D).

#### Distribution

Russia, Siberia, Far East (Teslenko and Zhiltzova 2009). Mongolia, North Korea, Japan (DeWalt et al. 2024). The genus *Megarcys* Klapálek, 1912 and species *M.ochracea* are recorded for China for the first time.

#### Notes

Shi (2022) regarded this genus and species as new records from Jiling Province (Mt. Changbai) in his Master’s degree thesis (data are available from CNKI), but it was not published.

### Utaperla lepnevae

(Zhiltzova, 1970)

E8618020-2F08-5FAD-ACC3-A986B9A352A9

Paraperla lepnevae Zhiltzova & Levanidova, 1970: 380.
Utaperla lepnevae Stark, Baumann, Kondratieff & Stewart, 2013: 101.

#### Materials

- **Type status:** Other material. **Occurrence:** individualCount: 2; sex: 2 males; lifeStage: adult; occurrenceStatus: present; occurrenceID: 0E458D34-9030-5DF9-B810-94A33F22B09B; **Taxon:** scientificName: *Utaperlalepnevae* (Zhiltzova, 1970); kingdom: Animalia; phylum: Arthropoda; class: Insecta; order: Plecoptera; family: Chloroperlidae; genus: Utaperla; specificEpithet: *lepnevae*; taxonomicStatus: valid; **Location:** continent: Asia; country: China; countryCode: CN; stateProvince: Heilongjiang; locality: Da Hinggan Ling Prefecture, Jiagedaqi District; maximumElevationInMeters: 451; verbatimLatitude: 50.403951; verbatimLongitude: 124.120544; **Identification:** identifiedBy: Abdur Rehman & Valentina A. Teslenko; **Event:** year: 2023; month: 6; day: 3; **Record Level:** language: en; institutionCode: ICYZU
- **Type status:** Other material. **Occurrence:** individualCount: 1; sex: 1 males; lifeStage: adult; occurrenceStatus: present; occurrenceID: 0E458D34-9030-5DF9-B810-94A33F22B09B; **Taxon:** scientificName: *Utaperlalepnevae* (Zhiltzova, 1970); kingdom: Animalia; phylum: Arthropoda; class: Insecta; order: Plecoptera; family: Chloroperlidae; genus: Utaperla; specificEpithet: *lepnevae*; taxonomicStatus: valid; **Location:** continent: Asia; country: China; countryCode: CN; stateProvince: Heilongjiang; locality: Da Hinggan Ling Prefecture, Xinlin District, Dawusu town; maximumElevationInMeters: 451; verbatimLatitude: 51.735212; verbatimLongitude: 124.504425; **Identification:** identifiedBy: Abdur Rehman & Valentina A. Teslenko; **Event:** year: 2023; month: 6; day: 5; **Record Level:** language: en; institutionCode: ICYZU
- **Type status:** Other material. **Occurrence:** individualCount: 18; sex: 8 males, 10 females; lifeStage: adult; occurrenceStatus: present; occurrenceID: 0E458D34-9030-5DF9-B810-94A33F22B09B; **Taxon:** scientificName: *Utaperlalepnevae* (Zhiltzova, 1970); kingdom: Animalia; phylum: Arthropoda; class: Insecta; order: Plecoptera; family: Chloroperlidae; genus: Utaperla; specificEpithet: *lepnevae*; taxonomicStatus: valid; **Location:** continent: Asia; country: China; countryCode: CN; stateProvince: Heilongjiang; locality: Da Hinggan Ling Prefecture, Xinlin District, Taergen town; maximumElevationInMeters: 392; verbatimLatitude: 52.232944; verbatimLongitude: 124.689441; **Identification:** identifiedBy: Abdur Rehman & Valentina A. Teslenko; **Event:** year: 2023; month: 6; day: 6; **Record Level:** language: en; institutionCode: ICYZU
- **Type status:** Other material. **Occurrence:** individualCount: 34; sex: 18 males , 16 females; lifeStage: adult; occurrenceStatus: present; occurrenceID: 0E458D34-9030-5DF9-B810-94A33F22B09B; **Taxon:** scientificName: *Utaperlalepnevae* (Zhiltzova, 1970); kingdom: Animalia; phylum: Arthropoda; class: Insecta; order: Plecoptera; family: Chloroperlidae; genus: Utaperla; specificEpithet: *lepnevae*; taxonomicStatus: valid; **Location:** continent: Asia; country: China; countryCode: CN; stateProvince: Heilongjiang; locality: Da Hinggan Ling Prefecture, Mohe county, Amuer town; maximumElevationInMeters: 451; verbatimLatitude: 52.833932; verbatimLongitude: 123.173923; **Identification:** identifiedBy: Abdur Rehman & Valentina A. Teslenko; **Event:** year: 2023; month: 6; day: 8; **Record Level:** language: en; institutionCode: ICYZU
- **Type status:** Other material. **Occurrence:** individualCount: 1; sex: 1 males; lifeStage: adult; occurrenceStatus: present; occurrenceID: 0E458D34-9030-5DF9-B810-94A33F22B09B; **Taxon:** scientificName: *Utaperlalepnevae* (Zhiltzova, 1970); kingdom: Animalia; phylum: Arthropoda; class: Insecta; order: Plecoptera; family: Chloroperlidae; genus: Utaperla; specificEpithet: *lepnevae*; taxonomicStatus: valid; **Location:** continent: Asia; country: China; countryCode: CN; stateProvince: Heilongjiang; locality: Da Hinggan Ling Prefecture, Xinlin District, Dawusu town; maximumElevationInMeters: 500; verbatimLatitude: 51.735212; verbatimLongitude: 124.504425; **Identification:** identifiedBy: Abdur Rehman & Valentina A. Teslenko; **Event:** year: 2023; month: 6; day: 5; **Record Level:** language: en; institutionCode: ICYZU

#### Diagnosis

**Figure 7.**
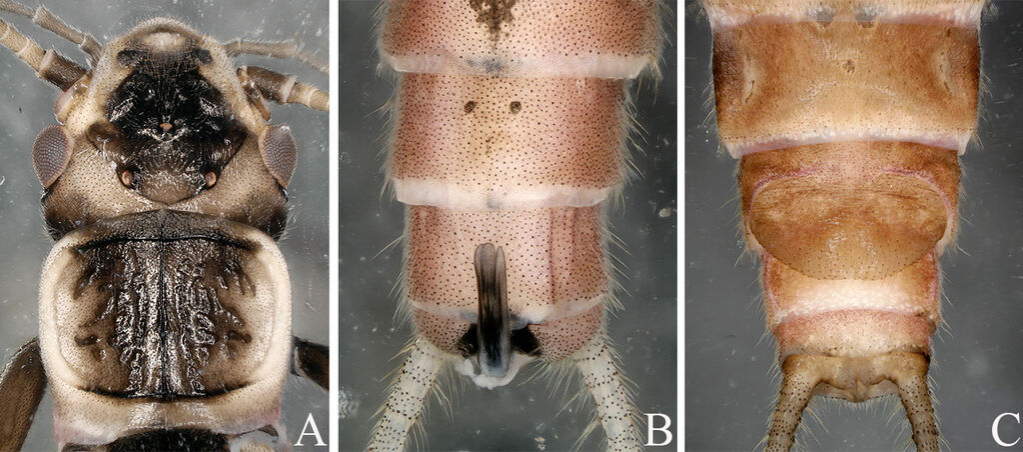
*Utaperlalepnevae* (Zhiltzova, 1970). **A** male head and pronotum; **B** male terminalia, dorsal view; **C** female terminalia, ventral view.

The head is elongated behind the eyes, the postfrontal suture is triangular, the ocelli are equidistant from each other. The epiproct of the male consists of three plates. The subgenital plate of female is very wide at the base, tapering posteriorly (Fig. 7A-C).

#### Distribution

Russia, Far East (Teslenko and Zhiltzova 2009). The species is recorded for China for the first time.

## Discussion

This study further elucidated the species diversity and faunal composition of stoneflies in the Greater Khingan Mountains of China and updated the distribution of stoneflies in the north-eastern Palaearctic Region. The research findings indicate that the stonefly fauna composition in the Greater Khingan Mountains is closely linked to neighbouring areas of the Russian Far East and Mongolia. Populations of some species may exist in isolated distributions. Especially in north-eastern and north-western China, there are still many regions awaiting further investigation in the future. Stoneflies spanning North America and Eurasia comprise 10 families and 46 genera (DeWalt et al. 2024). Through this study, nine families and 33 genera have been recorded in China (Yang and Li 2018). With further biodiversity surveys across northern China, it is highly probable that additional stonefly with Holarctic (or at least North Asian) distribution will be discovered in the future. These data will provide a basis for understanding the faunal characteristics of the local stonefly.

## Supplementary Material

XML Treatment for Isocapnia kudia

XML Treatment for Paraleuctra zapekinae

XML Treatment for Nemoura arctica

XML Treatment for Nemoura sirotskii

XML Treatment for Diura majuscula

XML Treatment for Megarcys ochracea

XML Treatment for Utaperla lepnevae
